# Ectopic Hepatocellular Carcinoma in the Gallbladder Wall With Choledocholithiasis: A Case Report

**DOI:** 10.7759/cureus.49534

**Published:** 2023-11-27

**Authors:** Takayoshi Nakajima, Tadashi Tsukamoto

**Affiliations:** 1 Department of Surgery, Meiwa Hospital, Nishinomiya, JPN; 2 Department of Hepato-Biliary-Pancreatic Surgery, Juso City General Hospital, Osaka, JPN

**Keywords:** cholecystectomy, choledocholithiasis, gallbladder, ectopic liver, ectopic hepatocellular carcinoma

## Abstract

An ectopic hepatocellular carcinoma (HCC) that originates from the ectopic liver is defined as a hepatic organ or tissue not conventionally linked to surrounding tissues. Ectopic HCC has a rare clinical incidence, and diagnosing it before surgery is challenging. Its characteristics and biological behavior have not been fully elucidated. This report presents a unique case of ectopic HCC in the gallbladder, discontinuous with the liver. A 74-year-old man was referred to our hospital after primarily complaining of fever and right hypochondrium pain. Plain computed tomography revealed a significantly thickened gallbladder wall containing fluid collection and incarceration of gallstone in the common bile duct. He was diagnosed with acute cholecystitis and obstructive cholangitis due to choledocholithiasis. After percutaneous transhepatic gallbladder drainage and endoscopic lithotomy for choledocholithiasis, a cholecystectomy was performed. Macroscopically, the resected gallbladder had a yellowish tumor (1 cm) within the significantly thickened gallbladder wall. Histopathological examination identified moderately differentiated HCC on the ectopic liver, discontinuous with the liver. Immunohistologically, the tumor was finally diagnosed as ectopic HCC with alpha-fetoprotein positive expression.

## Introduction

Liver tissue located outside the liver with a hepatic connection is typically referred to as an accessory liver, and that without a connection to the mother liver is called an ectopic liver tissue. Ectopic liver is usually asymptomatic, and its incidence has been documented to range from 0.24% to 0.47% in laparoscopy or autopsy [1,2]. It can manifest in different locations adjacent to the liver, such as the gallbladder, hepatic ligaments, omentum, retroperitoneum, pancreas, adrenal glands, and thorax [3,4].

An ectopic hepatocellular carcinoma (HCC) is defined as an HCC arising from hepatic parenchyma located in an extrahepatic organ or tissue [5]. It is a rare malignant disease, which exhibits morphological and immunohistochemical features closely resembling those of intrahepatic HCC [6]. The clinical features and management of ectopic HCC are not fully elucidated. Furthermore, limited reports of ectopic HCC arising from the gallbladder exist. We herein present a rare case of ectopic HCC in the gallbladder wall with choledocholithiasis.

## Case presentation

**Figure 1:**
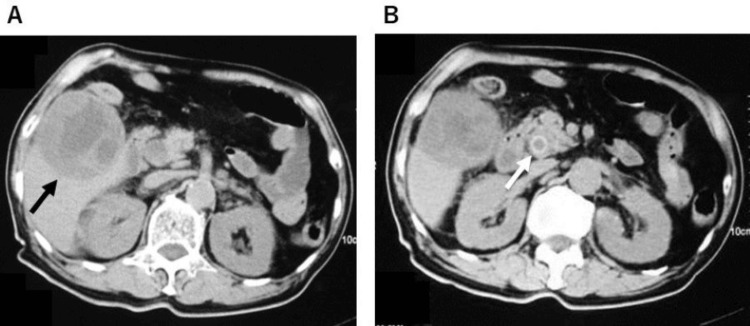
Pretreatment computed tomography images. (A) A significantly thickened gallbladder wall containing fluid collection (black arrow) and (B) incarceration of gallstone in the common bile duct (white arrow).

A 74-year-old Japanese male with a history of hypertension and angina pectoris was referred to our hospital after primarily complaining of fever and right hypochondrium pain. He had a personal history of social alcohol drinking of less than 40 g per day, and a smoking history of 15 cigarettes per day for 25 years but had quit for over 27 years. On abdominal examination, Murphy’s sign was positive. Laboratory tests were notable for a white blood cell count of 20.500/µL, aspartate transaminase of 114 U/L, alanine transaminase of 247 U/L, total bilirubin of 8.6 mg/dL, and C-reactive protein (CRP) of 22.14 mg/dL. He was uninfected with hepatitis virus such as hepatitis B virus and hepatitis C virus, and autoimmune tests showed no signs of positivity. Plain computed tomography of his abdomen revealed a significantly thickened gallbladder wall containing fluid collection and incarceration of gallstone in the common bile duct (Figure 1).

**Figure 2:**
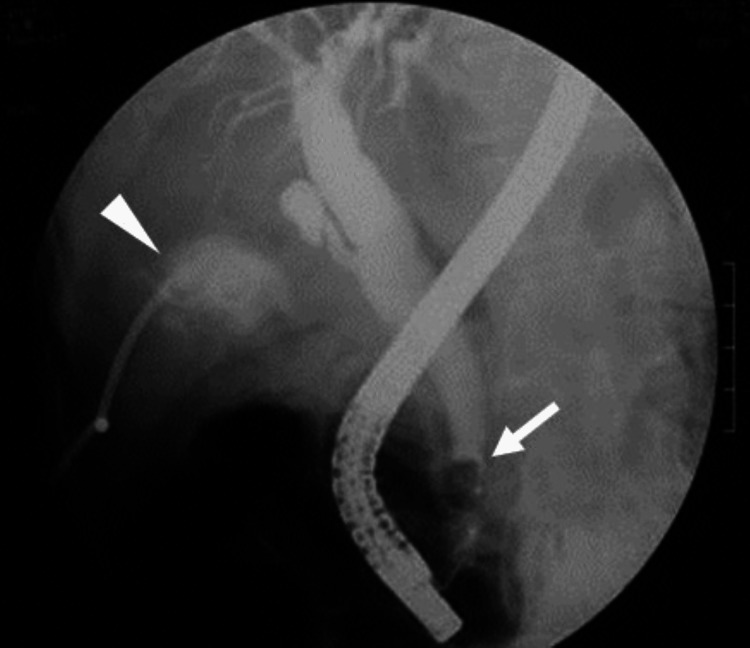
Percutaneous transhepatic gallbladder drainage and endoscopic lithotomy. A drainage tube was inserted into the gallbladder (arrowhead), and the gallstone in the common bile duct was endoscopically removed (arrow).

He was diagnosed with acute cholecystitis and obstructive cholangitis due to choledocholithiasis. Thus, emergent percutaneous transhepatic gallbladder drainage and endoscopic lithotomy were performed, along with antibiotic therapy. For cholecystitis, a 7.5 Fr drainage tube was inserted into the gallbladder, and 120 mL of bad smelling white-yellowish pus was aspirated. As a second procedure, the gallstone in the common bile duct was removed by endoscopic basket under the guidance of endoscopic retrograde cholangiography (Figure 2).

Following these procedures, his general condition dramatically improved. After the resolution of jaundice and the improvement of inflammatory markers, open cholecystectomy was performed. Intraoperatively, there were gangrenous changes in the inflamed gallbladder, and adhesions were noted between the gallbladder and omentum or duodenum. The liver exhibited macroscopic findings suggestive of alcoholic-related chronic hepatitis. We performed adhesolysis first by exposing Calot’s triangle, and a critical view of safety was maintained. After ligating the cystic artery and duct, the gallbladder was completely dissected off the cystic plate.

**Figure 3:**
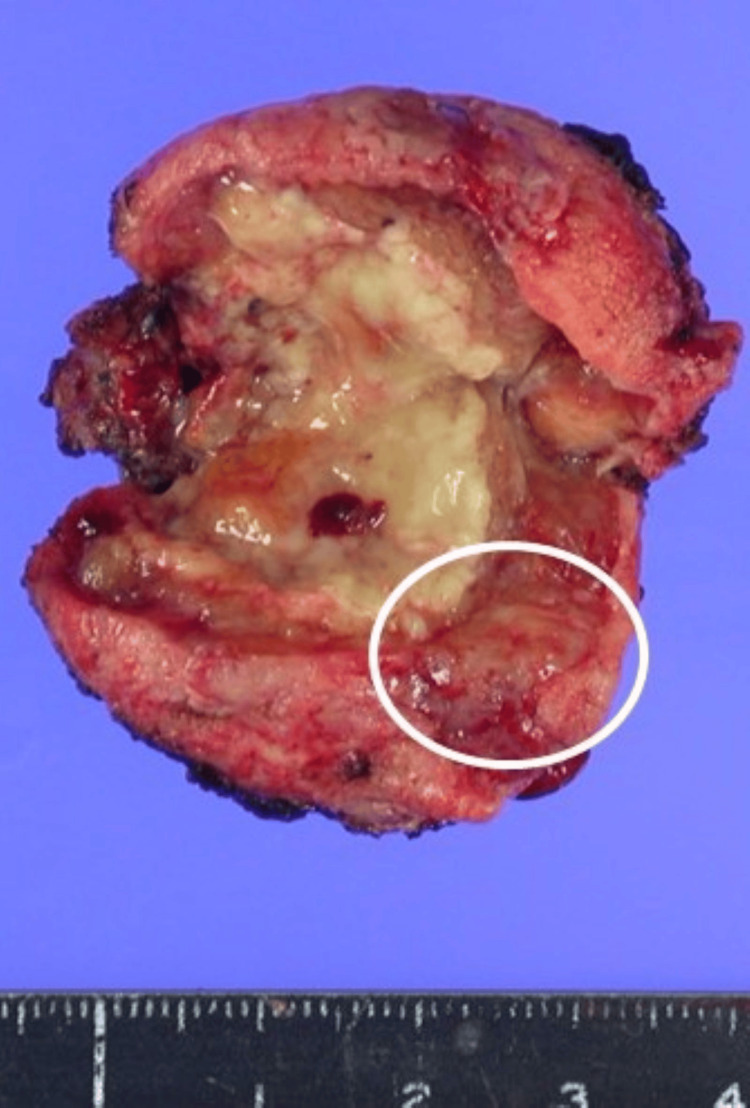
Resected specimen. A yellowish tumor (circle) was detected within the significantly thickened gallbladder wall.

Macroscopically, the resected gallbladder was necrotic and had severe inflammatory cell infiltration. A yellowish tumor (1 cm) was detected within the significantly thickened gallbladder wall (Figure 3).

**Figure 4:**
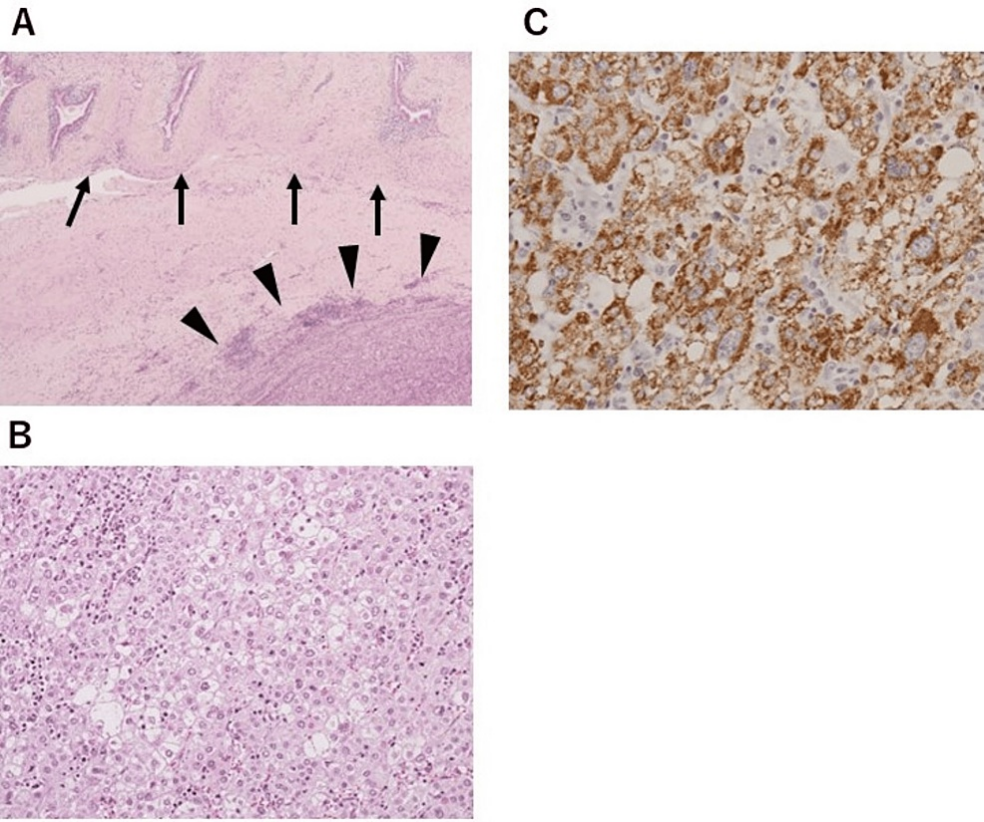
Histological and immunohistochemical findings. (A) The tumor (arrowheads) covered by connective tissue was within the gallbladder wall (arrows). (B) The tumor cells exhibited growth in irregular cords or plates separated by dilated sinusoidal vessels and had large hyperchromatic nuclei with prominent nucleoli and granular eosinophilic cytoplasm. (C) On immunohistochemical staining of the tumor, alpha-fetoprotein was positive.

Histologically, the tumor was covered by connective tissue, and no continuity with the liver parenchyma was observed (Figure 4A). The tumor cells exhibited growth in irregular cords or plates separated by dilated sinusoidal vessels (resembling a trabecular pattern). They had large hyperchromatic nuclei with prominent nucleoli and granular eosinophilic cytoplasm (Figure 4B). On immunohistochemical staining of the tumor, Hep Par-1 and Glypican 3 were negative, but alpha-fetoprotein was positive (Figure 4C).

From all the above, a diagnosis of moderately differentiated HCC within the gallbladder wall was made. Postoperatively, the patient did well and was discharged eleven days after surgery, and he remained asymptomatic for over five years after surgery with normal alpha-fetoprotein levels and no radiological signs of recurrence.

## Discussion

Ectopic HCC manifests morphological and immunohistochemical features closely resembling those of intrahepatic HCC. It is occasionally discovered by incidental imaging, and the diagnosis is difficult to confirm preoperatively [1,2,6]. Ectopic liver shares the same hepatocarcinogenesis risk factors as the liver, yet it appears that factors such as HBV, HCV, or liver cirrhosis are not implicated in its pathogenesis, as in our case [7]. Indeed, of the 24 ectopic HCC cases described by Adachi et al., only eight (33.3%) cases were infected with hepatitis virus [6]. The precise reason why ectopic liver is particularly predisposed to carcinogenesis remains unclear. It has been hypothesized that ectopic liver has a unique environment with a defective arterial supply and an absent connection with the portal and biliary systems, which can lead to exposure to carcinogens or damage to nuclear repair mechanisms, resulting in carcinogenesis [3,6,8].

Ectopic liver tissue is most frequently found in the gallbladder, even though it has also been reported in other locations such as the pancreas, spleen, hepatic ligaments, omentum, retroperitoneum, pancreas, adrenal glands, and thorax [3,4,6]. On the other hand, a comprehensive literature review of ectopic liver tissue located on the gallbladder surface or mesentery detected only two (0.04%) patients with ectopic liver tissue among 4,500 patients who underwent living donor hepatectomy or recipient hepatectomy. This review further noted that ectopic liver located outside the gallbladder had a higher risk of hepatocellular carcinoma, which is not reflected in the statistical analysis results [9]. The diagnosis of ectopic liver tissue additionally appears to occur when the patient has other medical conditions such as gallbladder stones and other biliary tract diseases [1,2,9]. Although the occurrence of ectopic liver tissue within the gallbladder is rare, it is important to be aware of this possibility.

We searched keywords ''ectopic hepatocellular carcinoma'' and ''gallbladder'' in PubMed and found four case reports of ectopic HCC originating from the gallbladder in full text [3,10-12]. No cases were preoperatively diagnosed with ectopic HCC. In two out of four cases, cholecystectomy was performed under suspicion of gallbladder cancer, leading to a histopathological diagnosis of ectopic HCC. To our knowledge, we are the first to report on ectopic HCC arising from the gallbladder wall with choledocholithiasis. The hypothesis that inflammatory thickening of the gallbladder wall due to cholecystitis caused by choledocholithiasis leads to carcinogenesis of the ectopic liver cannot be denied.

From a prognostic perspective, patients with ectopic HCC often experience a markedly more favorable clinical prognosis and lower recurrence rates compared to those with ordinary HCC, and surgical tumor removal frequently results in a complete excision [13,14]. Even in cases of ectopic HCC in the gallbladder, it is likely that a good prognosis can be expected if curative resection is achieved. The presence of ectopic liver during cholecystectomies performed for other reasons, or more rarely due to its clinical suspicion from preoperative imaging modalities, makes excision preferable given its significant carcinogenic potential.

## Conclusions

Ectopic liver is a rare condition resulting from abnormal embryological development of the liver that is usually asymptomatic. It may carry an increased risk of malignant degeneration to HCC, and the preoperative diagnosis of ectopic HCC is often very difficult. Therefore, ectopic liver should be recognized (including at cholecystectomy), removed, and examined histologically, even if there are no signs of chronic liver disease. Prompt surgical intervention for ectopic HCC may lead to a more favorable long-term prognosis.
